# Low-Temperature and High-Efficiency Solid-Phase Amplification Based on Formamide

**DOI:** 10.3390/mi15050565

**Published:** 2024-04-26

**Authors:** Jialing Huang, Huan Li, Fengfeng Shu, Wenchao Zhou, Yihui Wu, Yue Wang, Xiao Lv, Ming Gao, Zihan Song, Shixun Zhao

**Affiliations:** 1 School of Ophthalmology & Optometry, Wenzhou Medical University, Wenzhou 325035, China; 2 Changchun Institute of Optics, Fine Mechanics and Physics (CIOMP), Chinese Academy of Sciences, Changchun 130033, China; 3 University of Chinese Academy of Sciences, Beijing 100049, China

**Keywords:** formamide, melting temperature, immobilized primer, thermal stability, bridge amplification, reaction automation

## Abstract

The thermal stability of DNA immobilized on a solid surface is one of the factors that affects the efficiency of solid-phase amplification (SP-PCR). Although variable temperature amplification ensures high specificity of the reaction by precisely controlling temperature changes, excessively high temperatures during denaturation can negatively affect DNA stability. Formamide (FA) enables DNA denaturation at lower temperatures, showing potential for SP-PCR. Research on FA’s impacts on DNA microarrays is still limited, necessitating further optimization in exploring the characteristics of FA in SP-PCR according to particular application needs. We immobilized DNA on a chip using a crosslinker and generated DNA microarrays through bridge amplification based on FA denaturation on our automated reaction device. We optimized the denaturation and hybridization parameters of FA, achieving a maximum cluster density of 2.83 × 10^4^ colonies/mm^2^. Compared to high-temperature denaturation, FA denaturation required a lower template concentration and milder reaction conditions and produced higher cluster density, demonstrating that FA effectively improves hybridization rates on surfaces. Regarding the immobilized DNA stability, the FA group exhibited a 45% loss of DNA, resulting in a 15% higher DNA retention rate compared to the high-temperature group, indicating that FA can better maintain DNA stability. Our study suggests that using FA improves the immobilized DNA stability and amplification efficiency in SP-PCR.

## 1. Introduction

Solid-phase amplification (SP-PCR) is a technique that immobilizes one or two primers on a solid support, allowing DNA amplification on the surface. SP-PCR offers significant advantages over liquid-phase amplification, such as the ability to detect trace samples [1] and the immobilization of products on the surface, facilitating the detection of results. In addition, the immobilization of primers significantly minimizes false positive signals caused by primer dimerization [2,3,4]. These advantages give SP-PCR great application prospects in the fields of high-throughput gene sequencing [5], single nucleotide polymorphism (SNP) [6], and multiplex diagnosis [7].

Traditional SP-PCR typically relies on high-temperature denaturation, which leads to a decrease in surface DNA density [8,9], reducing the efficiency of SP-PCR. Over the years, researchers have immobilized DNA on the solid support through covalent coupling. However, even with the use of various immobilization methods such as 1-ethyl-3-(3-dimethylaminopropyl)-carbodiimide (EDC), benzene-1,3,5-triacetic acid (BTA), and various sulfonated analogues, the problem of heat-induced DNA loss after thermal cycling remains common. These methods lead to a loss of 40–60% of DNA after multiple thermal cycles [5,10], and even significant DNA loss can be observed in a short period of time [9,11]. Faced with this challenge, our research aims to study other gentler methods to lower the overall temperature of the reaction and improve the issue of DNA loss in SP-PCR, thereby enhancing the efficiency and accuracy of SP-PCR.

Formamide (FA) is a chemical reagent that denatures the secondary structure of DNA by generating hydrogen bond coupling with the DNA bases, thus lowering the melting temperature (Tm) of DNA [12,13]. Therefore, it is often used for DNA denaturation in various hybridization techniques, such as fluorescence in situ hybridization [14,15] and blot hybridization [16]. The conventional denaturation temperatures of PCR are typically between 94 °C and 98 °C, but FA can reduce them to 80 °C or even lower due to its hydrogen bond-breaking properties [17]. The fact that DNA can be amplified at comparatively lower temperatures suggests a reduced risk of heat-induced DNA loss and degradation. This may have a positive effect on SP-PCR, particularly given that the immobilized primers exhibit greater stability in a milder environment. This enhanced stability can significantly contribute to improving the efficiency of the amplification process. Currently, research mainly focuses on how FA affects DNA stability in liquid-phase environments, while there are relatively few studies on its effects on a DNA microarray, and these studies have several limitations [18,19]. Specifically, these studies only explored the effects of FA at concentrations below 20% and 45%, and mostly employed shorter oligonucleotides as samples. Furthermore, they did not fully account for the potential impact of solid surface effects on immobilized DNA. Given that the extent of FA’s effect on the Tm is closely related to the (G+C) composition and helical structure of DNA [13], these limitations mean that their conclusions may not apply to other various experimental platforms, particularly when it comes to long strands of DNA with complex (G+C) compositions and solid surfaces with different chemical properties. Hence, a thorough and systematic study is still needed to solidify the theoretical foundation for real-world use and enhance comprehension of FA’s influence on immobilized DNA on the surfaces within the specified experimental settings.

This study explored the promoting effects of FA in SP-PCR. Primers were immobilized on the amino surface of the microfluidic chip using zero-length crosslinker 1-ethyl-3-(3-dimethyl-aminopropyl) carbodiimide (EDC)/1-methylimidazole chemistry. Then, DNA microarrays were generated through bridge amplification on the automated device, utilizing both FA denaturation and high-temperature denaturation. We conducted a series of gradient experiments to optimize the denaturation parameters and hybridization conditions of FA. By comparing the results with those obtained from the high-temperature group, we explored the promoting effects of FA on surface hybridization. Furthermore, we compared the thermal stability of immobilized DNA under optimized conditions for both methods and discussed the significance of FA’s role in preserving DNA stability. This study is expected to provide some valuable insights for improving DNA stability and amplification efficiency in SP-PCR.

## 2. Materials and Methods

### 2.1. Reagents and Instruments

These included 1-ethyl-3-(3-dimethylaminopropyl)-carbodiimide (EDC) and 1-methylimidazole (Im) (Macklin Biochemical, Shanghai, China); 20× SSC buffer (GenClone Biotechnology, Beijing, China); 2× Phanta Max Master Mix, VAHTS Universal DNA Library Prep Kit for Illumina V3/ND607, VAHTS HiFi Amplification Mix/N616, VAHTS DNA Adapters for Illumina/N805, VAHTS DNA Clean Beads, Taq DNA polymerase, dNTP Mix (Vazyme Biotech, Nanjing, China); 10,000× SYBR Green I, Betaine and Bovine Serum Albumin (Solarbio, Beijing, China); formamide (Yuanye, Shanghai, China); OP AminoSlideTM (CapitalBio Technology, Chengdu, China); Qubit 1X dsDNA HS Kit, Qubit™ 4 Fluorometer, ProFlex™ 96-Well PCR System (Thermo Fisher Scientific, Waltham, MA, USA); and Agilent 2100 Bioanalyzer System (Agilent, Santa Clara, CA, USA).

### 2.2. Reaction Device

**Figure 1:**
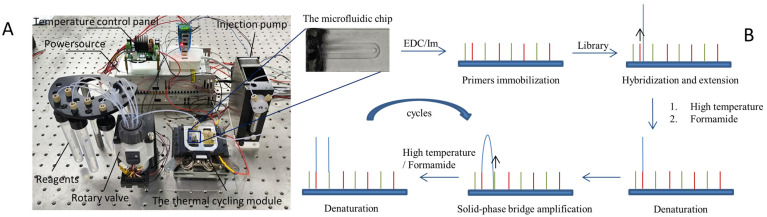
Diagram of the reaction device and experimental flowchart. (**A**) Diagram of the SP-PCR reaction device. The microfluidic chip used in the experiment features a U-shaped glass channel measuring 20 mm in length, 1.2 mm in width, and 0.5 mm in height. Its substrate is a glass slide called the OP AminoSlide, which is modified with amino groups. (**B**) Experimental flowchart. Firstly, the primers are immobilized on the chip using the crosslinker, EDC. After that, the library is hybridized and extended, followed by SP-PCR. There are two denaturation techniques used in the amplification process: formamide denaturation and high-temperature denaturation.

The device utilized in this study comprises two principal components: a thermal cycling module and a microfluidic module, as depicted in Figure 1A. The thermal cycling module mainly consists of a Peltier-based semiconductor heating module, in addition to a temperature control panel, a temperature sensor, a radiator, and a copper block. On the other hand, the microfluidic module is made up of three parts: reagent storage chambers, a rotary valve, and an injection pump. The rotary valve alternates among multiple valves to pull the required reagents into the channel for reaction, while the injection pump generates negative pressure to drive the system. Using LabView 2017, this device regulates the fluid route and temperature. The experiment utilizing this device for primer immobilization and amplification was depicted in a flowchart, as shown in Figure 1B.

### 2.3. Synthetic Oligonucleotides

In this study, immobilized primers were designed based on the sequence of the adapters used for library construction, ensuring complementarity with a portion of the library sequence during bridge amplification. The primers were designed as follows:

Primer-P1/P3: 5′-TTTTTTTTTTCAAGCAGAAGACGGCATACG-3′;
Primer-P2/P4: 5′-TTTTTTTTTTAATGATACGGCGACCACCGAGA-3′;

To minimize the steric hindrance on the solid surface, we have added a 10-T sequence to the 5′ position. The 5′ ends of all primers were modified with phosphate groups (5′-P) to form the phosphoramidate bonds with amino groups on the solid surface. The primers P1 and P2, with their 3′ ends remaining unmodified, functioned as forward and reverse primers for SP-PCR. In order to assess the stability of immobilized primers, fluorescent groups (3′-Cy3) were added to the 3′ ends of primers P3 and P4. They were all synthesized by Sangon Biotech (Shanghai) Co., Ltd. (Shanghai, China).

### 2.4. Library Preparation

The samples were deep-sea microorganisms provided by the Institute of Deep-sea Science and Engineering, Chinese Academy of Sciences, and their genomes were extracted by our colleagues. The V3–V4 region was cloned from the extracted genome using the following PCR mix solution: 2× Phanta Max Master Mix, 0.4 µM forward and reverse primers, and 150 ng DNA. The thermocycling process consisted of the following steps: initial denaturation at 95 °C for 3 min, followed by 30 cycles of denaturation at 95 °C for 15 s, annealing at 57 °C for 15 s, and extension at 72 °C for 30 s. At 72 °C, a 5 min extension step was then carried out. After that, the amplicons were processed for library preparation using the kits ND607, N616 and N805, and the product was characterized by the Qubit™ 4 Fluorometer and the Agilent 2100 Bioanalyzer.

### 2.5. Attachment of Oligonucleotides to Aminated Surface

The 5′ ends of primers P1 and P2 are modified with phosphate groups. In the presence of EDC and 1-methylimidazole, these phosphate groups can bind to the amino groups on the chip, forming phosphoramidate bonds. This consequently immobilizes the primers onto the chip. Primers P1 and P2 were prepared as a 1 µM solution in 10 mM 1-methylimidazole containing 40 mM EDC. The solution was pumped into the channel of the microfluidic chip and incubated at 50 °C for 60 min in a humid atmosphere to avoid evaporation. Following incubation, the channel was rinsed with 5× SSC buffer containing 0.1% Tween 20 for 2 min and rinsed and stored in 5× SSC buffer until used.

### 2.6. Solid-Phase Amplification on the Automated Device

The SP-PCR was first performed based on the method of high-temperature denaturation (high-temperature group). The chip was subjected to a 1 h blocking step with 0.1% BSA containing 5× SSC buffer and 0.1% Tween 20. Subsequently, it was rinsed with 5× SSC buffer and then deionized water sequentially. The PCR mix solution contains 1× PCR Buffer, 0.25 mM dNTPs, 2.5 mM MgCl_2_, 1 M betaine, 0.4 mg/mL BSA, 0.1% Tween 20, and 0.05 U/µL Taq DNA polymerase. The thermocycling was carried out as follows: denaturation at 95 °C for 1 min, annealing at 45 °C for 2 min and extension at 73 °C for 2 min. Fresh PCR mix was pumped in during the final 20 s of each cycle. This entire procedure was repeated for 40 cycles. At the end of the reaction, the chip was rinsed with 5× SSC buffer containing 0.1% Tween 20, 5× SSC buffer and water sequentially for 3 min each time. 

The thermocycling for SP-PCR based on FA denaturation (FA group) was carried out as follows: FA was introduced into the channel, followed by denaturation at 80 °C for 1 min. Subsequently, fresh PCR mix was pumped in, and then annealing was performed at 35 °C for 2 min, followed by extension at 73 °C for 2 min. The whole procedure was repeated for 40 cycles. All other treatments were the same as those in the high-temperature group.

### 2.7. Visualization of Clusters and Data Analysis

**Figure 2:**
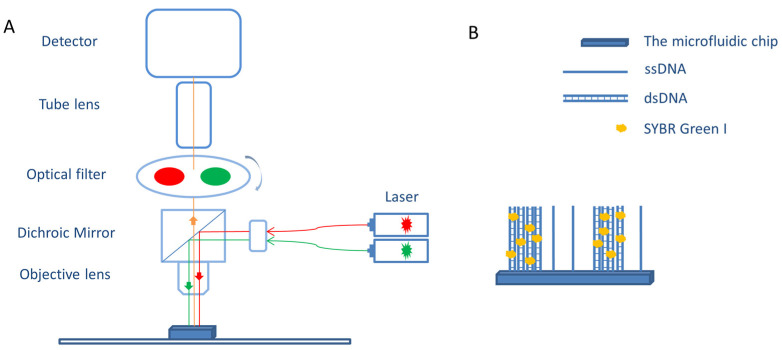
Imaging schematic. (**A**) The microscope detector was Andor’s EMCCD image sensor, and the excitation wavelength of the laser was 532 nm. (**B**) The amplification products were double-stranded DNA clusters immobilized on the surface, and SYBR Green I, which binds to these double-stranded structures, is used for their visualization.

Amplified DNA clusters were stained with a 1× SYBR Green I solution for 5 min. Images of the clusters were captured by a self-made fluorescent microscope, and the schematic diagram of the imaging process is shown in Figure 2. Finally, the density of the clusters was statistically analyzed by ImageJ (version 1.8.0).

### 2.8. Evaluation of the Thermal Stability of the Immobilized Primers

Primers P3 and P4 were immobilized on the chip, and thermocycling reactions based on high-temperature denaturation and FA denaturation were performed, respectively, according to the steps mentioned above. Each treatment was repeated multiple times independently. Following the reaction, the same course of treatment was taken. The results were characterized using our fluorescence microscope. Lastly, ImageJ was used to analyze fluorescence intensity on the chips, followed by calculating the mean intensity, and standard deviation. Additionally, the Wilcoxon rank-sum test was performed to assess the statistical significance of the difference between two treatments.

## 3. Results and Discussion

### 3.1. Library Validation

**Figure 3:**
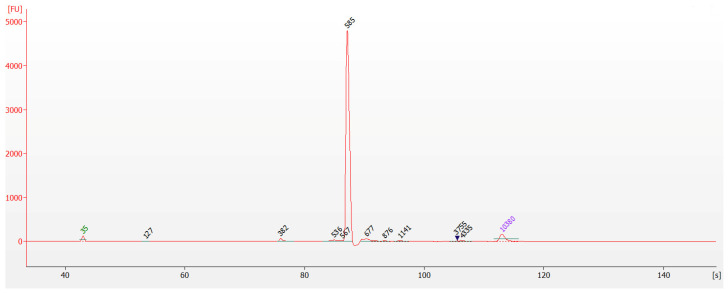
Result of capillary electrophoresis for library.

The library concentration was determined to be 61.8 ng/µL by the Qubit™ 4 Fluorometer. It was then diluted to less than 10 ng/µL and identified for fragment size through capillary electrophoresis. As shown in Figure 3, the library fragments were approximately 590 bp, as per our expectations. The concentration of target fragments was exceptionally high, accounting for a large proportion of the system, while the content of other non-target fragments was negligible. This purified product was suitable for utilization in the subsequent step of the experiment.

### 3.2. SP-PCR Based on High-Temperature Denaturation

**Figure 4:**
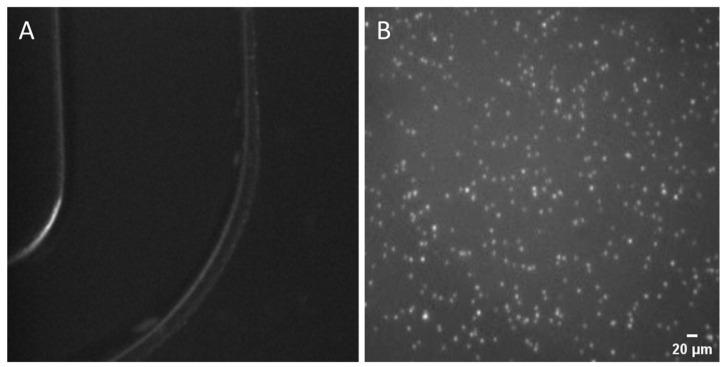
Solid-phase amplification based on high-temperature denaturation. The library concentration was 40 pM, and the annealing temperature was 45 °C. (**A**) The channel of the microfluidic chip. (**B**) The clusters are clearly visible, and their diameters range from approximately 2.5 to 4 µm.

Firstly, we conducted an SP-PCR based on high-temperature denaturation to confirm that the experiment’s fundamental design complied with SP-PCR specifications. The result is shown in Figure 4, where the clusters displayed a high brightness and a uniform dispersion on the chip’s surface, with diameters ranging from roughly 2.5 to 4 µm. The signal-to-noise ratio (SNR) of the clusters ranged from 2 to 5. These findings initially demonstrated the success of EDC/1-methylimidazole covalent crosslinking chemistry in immobilizing the primers onto the surface, laying a foundation for our future SP-PCR. Furthermore, they also exhibited that our thermal cycling module fulfills the basic requirements for PCR amplification, particularly in terms of temperature stability and heating/cooling rates. Additionally, the self-made microfluidic chip enabled efficient amplification, attributed to the optimized PCR mix solution and precise control over temperature and time. The distribution and morphology of the clusters can be clearly observed from the image, which further verifies the accuracy and reliability of the optical imaging system in result detection. In summary, the methods we employed for immobilizing primers on the chip, the PCR mix solution, the reaction, and imaging system have all proved to be feasible. Subsequent work will then be carried out based on these experimental circumstances and parameters.

### 3.3. Optimization of Denaturation Conditions

#### 3.3.1. The Concentration of FA

**Figure 5:**
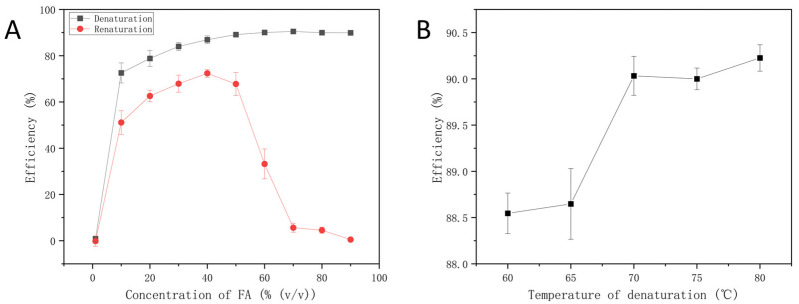
Optimization of denaturation conditions in SP-PCR. (**A**) The concentration of FA. Denaturation conditions: at 80 °C for 5 min; renaturation conditions: at 80 °C for 5 min, then cooled down to 40 °C and maintained for 30 min. (**B**) The denaturation temperature with 50% formamide. Denaturation conditions: 5 min at different temperature levels.

We evaluated the impact of FA concentrations on the denaturation and renaturation of the previously prepared library. The results were characterized by the Qubit 1× dsDNA HS Assay Kit and the Qubit™ 4 Fluorometer, as shown in Figure 5A. As the concentration of FA increased, the DNA stability was reduced and resulted in an increased denaturation efficiency. Within the range of 20–50%, the renaturation efficiency of DNA was relatively high. Nevertheless, the renaturation efficiency significantly dropped with additional increases in FA. FA is a stronger donor and acceptor than water, and most of its effects as a denaturant are related to the hydrogen bonds in DNA and DNA hydrates [13]. DNA unwinds when its stability is reduced due to a rise in the concentration of FA, which creates more binding sites for hydrogen bonds with DNA. In conclusion, 50% FA was chosen for denaturation because it can both ensure high denaturation efficiency and preserve a favorable renaturation effect.

#### 3.3.2. The Temperature of Denaturation

Typically, a precise concentration of FA is often present in DNA solutions, and this concentration effectively denatures DNA within a specified temperature range [17]. We evaluated how 50% FA affected DNA denaturation efficiency at various denaturation temperatures. As depicted in Figure 5B, DNA maintained a high level of denaturation efficiency of over 88%. Compared to conventional high-temperature denaturation methods, FA effectively breaks down hydrogen bond stability at lower temperatures, which speeds up the disintegration of the DNA helix. Given that FA exhibits the most prominent effect at 80 °C, we chose 80 °C as the denaturation temperature in SP-PCR.

### 3.4. Optimization of Hybridization Conditions

#### 3.4.1. The Annealing Temperature of SP-PCR

**Figure 6:**
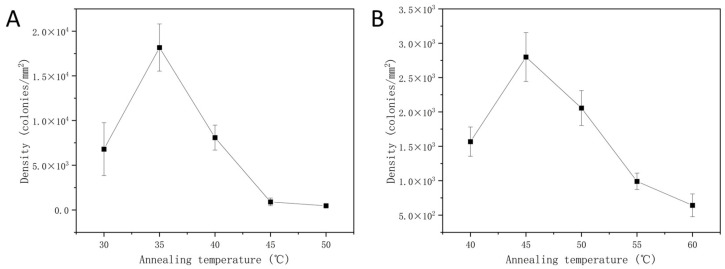

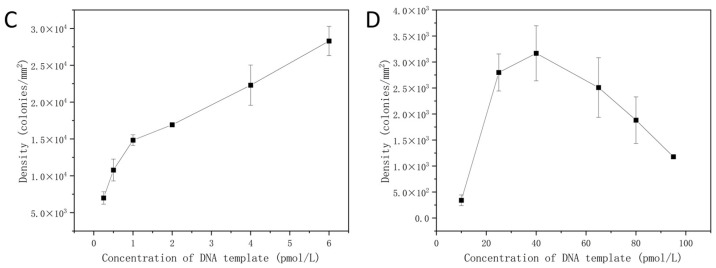
Optimization of hybridization conditions in SP-PCR. (**A**) The FA group, annealing temperature. (**B**) The high-temperature group, annealing temperature. (**C**) The FA group, library concentration. (**D**) The high-temperature group, library concentration. The scales of the vertical axis in (**B**,**D**) are roughly one-tenth of those in (**A**,**C**). In addition, the image results of the data shown above are provided in Figures S1 and S2 in the Supplementary Materials.

The kinetics of DNA template hybridization in the solid phase are affected by annealing temperatures. The evaluation of hybridization efficiency can be achieved by analyzing the density of clusters. Figure 6A,B depict the results of annealing temperatures for the FA group and the high-temperature group, respectively. Both results indicate that the density of clusters initially increased, peaking at 35 °C and 45 °C, respectively, before decreasing as the annealing temperature increased. Compared to the high-temperature group, we were able to achieve efficient DNA hybridization in a gentler environment by using FA. At the same time, the higher density produced by the FA group suggests that it allows more base-specific pairing even at comparatively low system temperatures.

#### 3.4.2. The Template Concentration of SP-PCR

**Figure 7:**
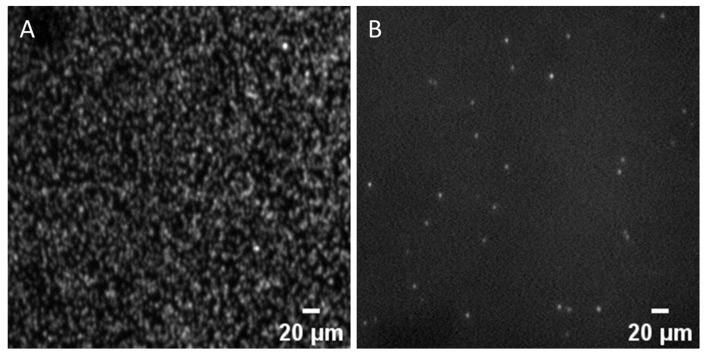
Solid-phase amplification. (**A**) Dense clusters were observed in SP-PCR based on FA denaturation, with 6 pM DNA template. (**B**) Sparse clusters were observed in SP-PCR based on high-temperature denaturation, with 6 pM DNA template.

The effect of FA on template hybridization efficiency was explored by setting up a template concentration gradient. Figure 6C,D display the results for the FA group and the high-temperature group, respectively. As the DNA template concentration rose in the FA group, so did the cluster density. The hybridization of DNA templates with immobilized primers was effective within the low concentration range (below 10 pM). As illustrated in Figure 7A, the cluster density achieved with 6 pM DNA templates was 2.83 × 10^4^ colonies/mm^2^ (We also performed an SP-PCR based on high-temperature denaturation with 6 pM DNA templates, and the outcome is displayed in Figure 7B). Since the clusters became too dense when the template concentration exceeded 6 pM, surpassing the resolution limit of the optical imaging system for individual clusters, no further increase in the DNA template concentration was attempted. In contrast, the cluster density in the high-temperature group initially increased and then fell as we increased the DNA concentration. At 40 pM, the hybridization efficiency reached its peak with a cluster density of 0.32 × 10^4^ colonies/mm^2^, and an inhibitory effect was observed thereafter. The primary reason for this difference is the inhibition of DNA secondary structure formation by FA, thereby lessening the competition between surface hybridization and secondary structures. As a result, the hybridization kinetics on the solid phase are somewhat improved, increasing the hybridization efficiency in SP-PCR.

### 3.5. Evaluation of the Thermal Stability of Immobilized Primers

**Figure 8:**
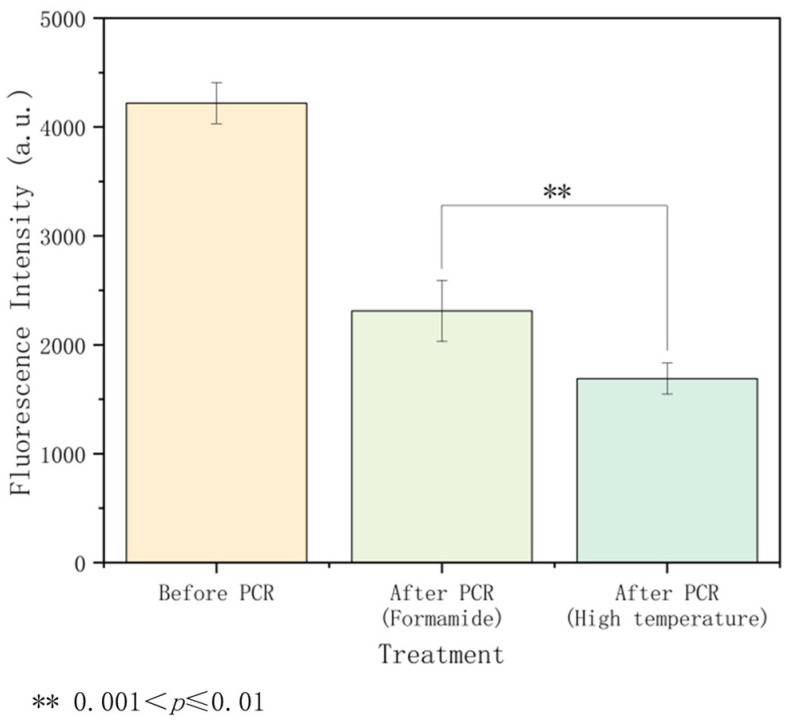
Evaluation of the thermal stability of immobilized primers. The 3′ ends of primers P3 and P4 were modified with Cy3 fluorescent groups, and the primers on the surface can be characterized by a fluorescence microscope (with a wavelength of 532 nm). Based on the statistical test, the *p*-value is less than 0.01, indicating a statistically significant difference between the two sets of data. The detailed data and statistical results are shown in Table S1 in the Supplementary Materials.

In this study, we validated the impact of temperature on immobilized DNA stability by testing the intensity of immobilized primers before and after the thermal cycling based on two denaturation methods: high temperature and FA. The results, presented in Figure 8, show that the DNA loss rate in the high-temperature group was nearly 60%, while the loss in the FA group was only 45%. The Wilcoxon rank-sum test revealed a p-value less than 0.01, indicating a statistically significant difference between the two groups. Taking into account the differences in Figure 6, we proposed that the high-temperature group disrupted the binding between primers and the surface during thermal cycling, increasing the distance between immobilized primers. This hindered the bridging of synthetic DNA with immobilized primers, resulting in lower amplification efficiency. In contrast, FA demonstrated significant benefits in preserving the stability of the immobilized primers. FA breaks the double-strand structure of DNA, but its effects are gentler and more controllable. The thermal effect on the immobilized primers was much reduced after high-temperature denaturation was substituted with FA. During SP-PCR, this increased stability showed up as a higher cluster density, suggesting that the amplification products were more uniform and dense. In conclusion, FA demonstrates better advantages in improving the stability of immobilized DNA through its unique denaturation mechanism. This improves the amplification’s efficiency and helps produce higher-quality products.

In this study, we used a denaturation temperature of 80 °C to guarantee effective DNA denaturation in solid-phase environments. The thorough denaturation of DNA provides ideal conditions for hybridization reactions, potentially enhancing hybridization efficiency. Furthermore, we have confirmed that reducing the temperature promotes DNA stability, thus providing a foundation for further research into the potential uses of lower denaturation temperatures in SP-PCR. Thus, we plan to explore SP-PCR based on lower denaturation temperatures in subsequent studies.

## 4. Conclusions

In summary, we have successfully achieved the automation of bridge amplification on our device and microfluidic chip, generating DNA microarrays. Firstly, we confirmed that effective SP-PCR can be performed on our thermal cycling/microfluidic device, which is based on the primer’s immobilization through EDC crosslinking chemistry. The clusters exhibited a uniform distribution, with diameters ranging from 2.5 to 4 µm and a signal-to-noise ratio of 2 to 5. This demonstrates the feasibility of our primer’s immobilization strategy, reaction settings, and the potential for automating SP-PCR on microfluidic chips using our reaction and detection device. Based on this experimental foundation, we optimized the use of formamide denaturation, replacing high-temperature denaturation. We determined that 80 °C was the optimal denaturation temperature for formamide. With a final concentration of 6 pM DNA library (diluting with 50% formamide) and annealing at 35 °C, we were able to obtain a maximum cluster density of 2.83 × 10^4^ colonies/mm^2^. Formamide denaturation exhibited higher sensitivity in SP-PCR, requiring a lower template concentration, operating at a more moderate temperature, and producing a higher cluster density than high-temperature denaturation. This demonstrated the advantageous effect of formamide on surface hybridization. Under optimized conditions, the immobilized DNA loss was 45% in formamide denaturation and 60% in high-temperature denaturation, suggesting that formamide’s capacity to lower temperature improves immobilized DNA stability more. Our results imply that formamide treatment helps preserve DNA stability on solid surfaces and improves the amplification efficiency in SP-PCR. Our knowledge of the formamide mechanism in SP-PCR has been enhanced by this work, which also offers valuable insights for enhancing immobilized DNA stability and amplification efficiency in real-world applications.

## Data Availability

The original contributions presented in the study are included in the article/Supplementary Materials, further inquiries can be directed to the corresponding authors.

## Supplementary Materials

The following supporting information can be downloaded at: https://www.mdpi.com/article/10.3390/mi15050565/s1, Figure S1: The image results corresponding to two sets of annealing temperature; Figure S2: The image results corresponding to two sets of library concentrations; Table S1: The statistical results of the thermal stability of immobilized primers.
