# Supplementary material for: Low-Temperature and High-Efficiency Solid-Phase Amplification Based on Formamide
Source: Micromachines (Basel). 2024 Apr 26;15(5):565. doi: 10.3390/mi15050565 (PMC11123353; doi:10.3390/mi15050565)
Supplement: Supplementary file 1 [file micromachines-15-00565-s001.zip › micromachines-2955328-supplementary.pdf]

Low-temperature and High-efficiency Solid-Phase Amplification Based on Formamide

Jialing Huang [1], Huan Li [2,*], Fengfeng Shu [2], Wenchao Zhou [2], Yihui Wu [1,2,*], Yue Wang [2], Xiao lv [2], Ming Gao [2,3], Zihan Song [2,3] and Shixun Zhao [2,3]

1 School of Ophthalmology & Optometry, Wenzhou Medical University, Wenzhou 325035, Zhejiang, China;

2 Changchun Institute of Optics, Fine Mechanics and Physics (CIOMP), Chinese Academy of Sciences, Changchun 130033, China;

3 University of Chinese Academy of Sciences, Beijing 100049, China

* Correspondence: lihuan@ciomp.ac.cn(H.L.); yihuiwu@ciomp.ac.cn(Y.W.); Tel.: +86-138-4310-1883(Y.W.); +86-135-9604-4762(H.L.)

1. Image results of the data

The image results of different annealing temperatures for the FA group and the high-temperature group are shown in Figure S1. Notably, a significant increase in cluster density was observed in the formamide group when annealed at 35°C. For the high-temperature group, similar densities were achieved at 45°C and 50°C. Statistical analysis indicated that the density was slightly higher at 45°C, supporting the choice of 45°C as the annealing temperature.

Based on the optimized annealing temperature, we conducted a test of library concentration, as illustrated in Figure S2. In the formamide group, the cluster density increased with concentration, with a notably denser cluster observed at 4 pM and 6 pM. In contrast, the cluster density in the high-temperature group was significantly sparser compared to the formamide group, peaking at approximately 40 pM.

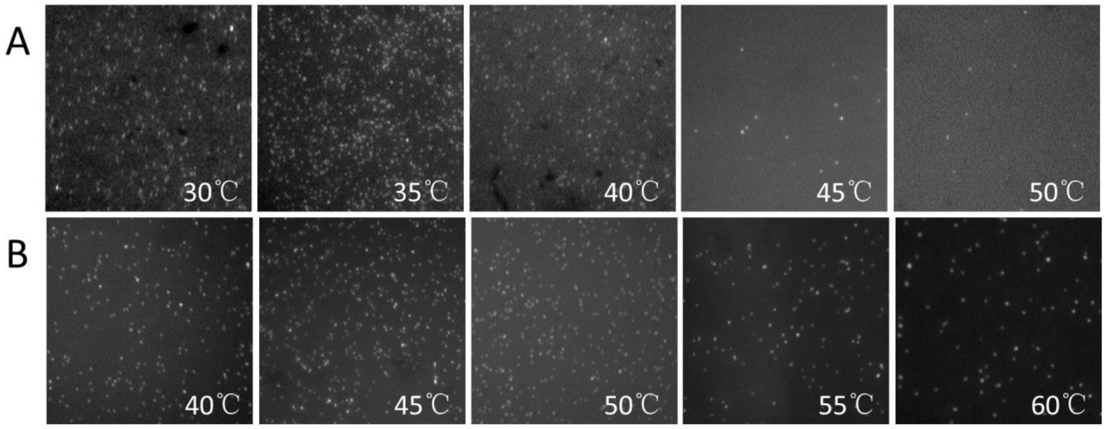

Figure S1. The image results corresponding to two sets of annealing temperatures. (A) Formamide group with a library concentration of 1 pM. (B) High-temperature group with a library concentration of 25 pM.

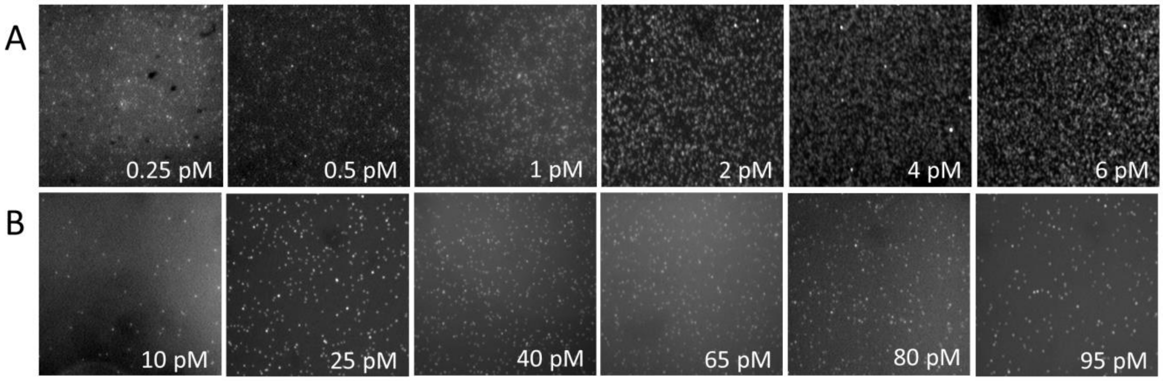

Figure S2. The image results corresponding to two sets of library concentrations. (A) Formamide group with an annealing temperature of 35°C. (B) High-temperature group with an annealing temperature of 45°C.

2. Statistical data of the thermal stability of immobilized primers

Table S1 presents the data on the thermal stability of immobilized primers. We analyzed the statistical differences between the formamide group and the high-temperature group using the Wilcoxon rank-sum test. With a P-value less than 0.01, we concluded that there was a significant statistical difference between the two groups of data.

Table S1. The statistical results of the thermal stability of immobilized primers.

Treatment	Fluorescence Intensity	N	Mean	SD	P
Before PCR	4023.636<br>4311.756<br>3839.757<br>4470.987<br>4260.286<br>4115.364<br>4438.568<br>4242.553<br>4271.647	9	4219.394889	187.9933737	
After PCR (Formamide)	1916.021<br>1917.064<br>1986.828<br>2471.579<br>2576.642<br>2576.268<br>2249.746<br>2507.071<br>2601.277	9	2311.388444	280.7638659	0.00391**
After PCR (High temperature)	1896.402<br>1849.699<br>1830.197<br>1743.373	9	1689.71356	143.30335	

1675.136
1602.176
1592.184
1434.02
1584.235

** $0.001 < p \leq 0.01$
